# Supplementary material for: The Venturia inaequalis effector repertoire is dominated by expanded families with predicted structural similarity, but unrelated sequence, to avirulence proteins from other plant-pathogenic fungi
Source: BMC Biol. 2022 Nov 3;20:246. doi: 10.1186/s12915-022-01442-9 (PMC9632046; doi:10.1186/s12915-022-01442-9)
Supplement: Supplementary file 1 — Additional file 1: Table S1. RNA-seq transcriptome sequencing read statistics from this study. Samples used for the RNA-seq transcriptome sequencing experiment were derived from an infection time course of Venturia inaequalis on detached leaves from susceptible apple cultivar ‘Royal Gala’ at 12 and 24 hours post-inoculation (hpi), as well as 2, 3, 5 and 7 days post-inoculation (dpi), and during growth. Table S2. Effector candidate (EC) protein families or singletons from Venturia inaequalis that have sequence similarity to EC or avirulence (Avr) effector proteins from other plant-pathogenic fungi. Table S3. Effector candidates (ECs) of Venturia inaequalis (Vi) with predicted structural similarity to EC or avirulence (Avr) effector proteins from other plant-pathogenic fungi that have a characterized tertiary structure present in the RCSB PDB. [file 12915_2022_1442_MOESM1_ESM.docx]

**Table S1** RNA-seq transcriptome sequencing read statistics from this study. Samples used for the RNA-seq transcriptome sequencing experiment were derived from an infection time course of *Venturia inaequalis* on detached leaves from susceptible apple cultivar ‘Royal Gala’ at 12 and 24 hours post-inoculation (hpi), as well as 2, 3, 5 and 7 days post-inoculation (dpi), and during growth of the fungus in culture on the surface of cellophane membranes overlying potato dextrose agar at 7 dpi.

| **Sample name** | **Time point** | **Tissue** | **Total number of reads** | **Number of paired reads mapped to the *V. inaequalis* MNH120 genome** | **Overall alignment rate** |
| --- | --- | --- | --- | --- | --- |
| S12V2 | 12 hpi | Apple leaf (*in planta*) | 72,515,775 | 91,069 | 0.13% |
| S12V3 |  |  | 71,722,781 | 84,531 | 0.12% |
| S12V4 |  |  | 65,009,930 | 78,419 | 0.12% |
| S12V5 |  |  | 61,088,214 | 123,211 | 0.21% |
| S24V1 | 24 hpi |  | 67,826,021 | 135,704 | 0.21% |
| S24V3 |  |  | 67,811,505 | 134,126 | 0.20% |
| S24V4 |  |  | 75,706,837 | 205,908 | 0.28% |
| S24V5 |  |  | 78,244,648 | 148,265 | 0.20% |
| S2V1 | 2 dpi |  | 47,424,653 | 125,061 | 0.27% |
| S2V2 |  |  | 68,132,940 | 279,525 | 0.42% |
| S2V3 |  |  | 73,356,846 | 327,163 | 0.46% |
| S2V5 |  |  | 63,149,333 | 136,993 | 0.22% |
| S3V1 | 3 dpi |  | 46,736,839 | 475,239 | 1.05% |
| S3V2 |  |  | 48,406,231 | 341,262 | 0.73% |
| S3V3 |  |  | 63,951,888 | 658,625 | 1.07% |
| S3V5 |  |  | 46,365,679 | 465,485 | 1.04% |
| S5V1 | 5 dpi |  | 40,513,300 | 1,439,733 | 3.68% |
| S5V2 |  |  | 36,798,369 | 494,654 | 1.39% |
| S5V3 |  |  | 53,569,444 | 2,965,281 | 5.73% |
| S5V4 |  |  | 46,221,769 | 1,666,765 | 3.73% |
| S7V2 | 7 dpi |  | 47,760,909 | 3,482,001 | 7.55% |
| S7V3 |  |  | 48,892,959 | 2,331,267 | 4.94% |
| S7V4 |  |  | 47,854,848 | 4,350,665 | 9.43% |
| S7V5 |  |  | 43,384,995 | 1,988,632 | 4.76% |
| SS1 | 7 dpi | In culture | 24,522,994 | 22,249,229 | 93.77% |
| SS3b |  |  | 20,754,255 | 18,915,521 | 93.99% |
| SS4b |  |  | 22,646,155 | 20,670,316 | 93.89% |
| SS8b |  |  | 26,675,000 | 24,347,911 | 94.34% |

**Table S2** Effector candidate (EC) protein families or singletons from *Venturia inaequalis* that have sequence similarity to EC or avirulence (Avr) effector proteins from other plant-pathogenic fungi.

| **EC protein family** | **Number of family members** | **Similar EC/Avr and host pathogen** | **Maximum amino acid identity to similar EC/Avr** | **Temporal gene expression wave** |
| --- | --- | --- | --- | --- |
| **Gas1-like family** | 2 | *Magnaporthe oryzae* Gas1 | 24.6% | Wave 2 (early infection) |
| **Ave1-like family** | 11 | *Verticillium dahliae* Ave1 | 36.5% | Waves 4 and 5 (mid-late infection) |
| **Ecp39-like family** | 6 | *Fulvia fulva* Ecp39 | 46.9% | Waves 4 and 5 (mid-late infection) |
| **Ecp10-like family** | 61 | *F. fulva* Ecp10 | 23.2% | Waves 4 and 5 (mid-late infection) |
| **AvrLm6-like family** | 31 | *Leptosphaeria maculans* AvrLm6 | 22.7% | Waves 4 and 5 (mid-late infection) |
| **Ecp6-like singleton** | NA | *F. fulva* Ecp6 | 43% | Wave 4 (mid-late infection) |

NA: Not applicable, as the protein is a singleton.

**Table S3** Effector candidates (ECs) of *Venturia inaequalis* (*Vi*) with predicted structural similarity to EC or avirulence (Avr) effector proteins from other plant-pathogenic fungi that have a characterized tertiary structure present in the RCSB PDB.

| *Vi* protein ID | EC family | Number of  family members | pLDDT score | Structural similarity (RCSB PDB ID) | Dali  Z-score | RMSD |
| --- | --- | --- | --- | --- | --- | --- |
| g13386 | Family 1 | 75 | 84.34 | *Magnaporthe oryzae* MAX effector (6R5J);  *M. oryzae* AvrPiz-t (2LW6); *Pyrenophora tritici-repentis* ToxB (2MM2);  *M. oryzae* Avr-Pia (2N37);  *M. oryzae* Avr-Pib (5Z1V); *M. oryzae* Avr1-CO39 (2MYV);  *M. oryzae* Avr-Pik (6FUB) | 4.9; 3.7;  3.4; 3.3; 3.3;  2.9;  2.4 | 3.81; 2.72;  2.98;  3.14;  4.71;  4.31;  3.95 |
| g11711 | Family 2 | 32 | 97.77 | *Zymoseptoria tritici* Zt-KP6-1 (6QPK) | 5.8 | 2.02 |
| g18375 | Family 5 | 36 | 89.01 | *Z. tritici* Zt-KP6-1 (6QPK) | 5.7 | 2.34 |
| g20030 | AvrLm6-like^1^ | 31 | 89.96 | *Z. tritici* Zt-KP6-1 (6QPK) | 4.2 | 2.79 |
| g4577 | Family 23 | 5 | 80.18 | *Z. tritici* Zt-KP6-1 (6QPK) | 5.9 | 3.03 |
| g12079 | Family 26 | 4 | 88.49 | *Z. tritici* Zt-KP6-1 (6QPK) | 4.9 | 3.15 |
| g18322 | Singleton | NA | 73.8 | *Z. tritici* Zt-KP6-1 (6QPK) | 4.4 | 3.28 |
| g4356 | Singleton | NA | 78.02 | *Z. tritici* Zt-KP6-1 (6QPK) | 5.8 | 2.57 |
| g4781 | Family 7 | 22 | 89.89 | *P. tritici-repentis* ToxA (1ZLE); *Fusarium oxysporum* Avr2/Six3 (5OD4); *Melampsora lini* AvrL567-A (2OPC) | 5.3;  3.2;  2.7 | 2.95;  3.63;  3.48 |
| g13172 | Family 28 | 6 | 59.95* | *P. tritici-repentis* ToxA (1ZLE); *F. oxysporum* Avr2/Six3 (5OD4); *M. lini* AvrL567-D (2QVT);  *M. lini* AvrL567-A (2OPC) | 7.4;  6.5; 5.3;  4.9 | 2.47;  3.36; 3.39;  3.78 |
| g9034 | Family 38 | 3 | 49.75 | *P. tritici-repentis* ToxA (1ZLE); *F. oxysporum* Avr2/Six3 (5OD4);  *M. lini* AvrL567-D (2QVT); *M. lini* AvrL567-A (2OPC) | 5.8;  5.0; 3.1;  2.9 | 3.01;  3.28; 3.39; 3.67 |
| g4288 | Singleton | NA | 55.02* | *F. oxysporum* Avr2/Six3 (5OD4);  *P. tritici-repentis* ToxA (1ZLE); *M. lini* AvrL567-A (2OPC) | 4.5;  3.6; 2.8 | 3.42;  3.38; 3.05 |
| g11097 | Family 15 | 12 | 89.58 | *Leptosphaeria maculans* AvrLm5-9 (7AD5);  *L. maculans* AvrLm4-7 (4FPR); *Fulvia fulva* Ecp11-1 (6ZUQ) | 6.5;  5.9;  5.4 | 3.50;  3.98;  3.63 |
| g24490 | Family 47 | 2 | 75.85 | *L. maculans* AvrLm4-7 (4FPR); *L. maculans* AvrLm5-9 (7AD5); *F. fulva* Ecp11-1 (6ZUQ) | 4.1;  3.6; 3.5 | 3.86;  3.61; 3.69 |
| g3787 | Family 49 | 3 | 78.15 | *F. oxysporum* Avr1/Six4 (7T6A);  *F. oxysporum* Avr3/Six2 (7T69) | NA;  NA | 5.10;  4.00 |

pLDDT: AlphaFold2 predicted Local Distance Difference Test score (0‒100). A score of 70–100 is indicative of medium to high confidence. RCSB PDB: Research Collaboratory for Structural Bioinformatics Protein Data Bank.

Z-score: A Dali Z-score above 2 indicates ‘significant similarities’ between proteins. RMSD: root-mean-square deviation (a measure of similarity between protein structures). The smaller the RMSD, the more similar the proteins structures are. NA: Not applicable, structures not available on Dali server database.

^1^: Effector candidate family has similarity to the AvrLm6 protein from *L. maculans.*

*: Indicates that the protein is predicted to have an intrinsically disordered region, which reduces the overall pLDDT score.
