# Supplementary material for: The Venturia inaequalis effector repertoire is dominated by expanded families with predicted structural similarity, but unrelated sequence, to avirulence proteins from other plant-pathogenic fungi
Source: BMC Biol. 2022 Nov 3;20:246. doi: 10.1186/s12915-022-01442-9 (PMC9632046; doi:10.1186/s12915-022-01442-9)
Supplement: Supplementary file 2 — Additional file 2: Fig. S1. Bioinformatic pipeline used for transcriptome analysis and genome annotation. Total RNA was extracted from apple leaves infected with Venturia inaequalis at 12 and 24 hours post-inoculation (hpi), as well as 2, 3, 5 and 7 days post-inoculation (dpi). As a reference for growth in culture, total RNA was also extracted from V. inaequalis grown on the surface of cellophane membranes overlaying potato dextrose agar at 7 dpi. Four biological replicates were included per sample. PE: paired-end; CDS: coding sequence; CAZyme: carbohydrate-active enzyme; EC: effector candidate. Fig. S2. Plant cell wall-degrading enzyme (PCWDE)-encoding genes of Venturia inaequalis upregulated during infection of susceptible apple cultivar ‘Royal Gala’, relative to growth of the fungus in culture on the surface of cellophane membranes overlying potato dextrose agar. A. Proportion of in planta upregulated PCWDE-encoding genes in each host infection-specific temporal expression wave. B. Heatmap of PCWDE-encoding genes upregulated in planta that demonstrate a peak level of expression during waves 1 and 2 of the early infection stage at 12 and 24 hours post-inoculation (hpi). C. Heatmap of PCWDE-encoding genes upregulated in planta that demonstrate a peak level of expression during wave 3 of the mid infection stage at 2 and 3 days post-inoculation (dpi) and waves 4 and 5 of the mid-late infection stage at 5 and 7 dpi. Block labels on the left indicate gene expression wave. Numbers in brackets indicate number of genes per wave. Gene expression data are scaled rlog-normalized counts across all samples (Z-score), averaged from four biological replicates. Labels on the right indicate carbohydrate-active enzyme (CAZyme) classification. Bar plots depict the maximum log2 DESeq2-normalized count value across all in planta time points. AA: auxiliary activity; GH: glycoside hydrolase; CE: carbohydrate esterase; PL: polysaccharide lyase; CBM: carbohydrate-binding module. Fig. S3. [file 12915_2022_1442_MOESM2_ESM.docx]

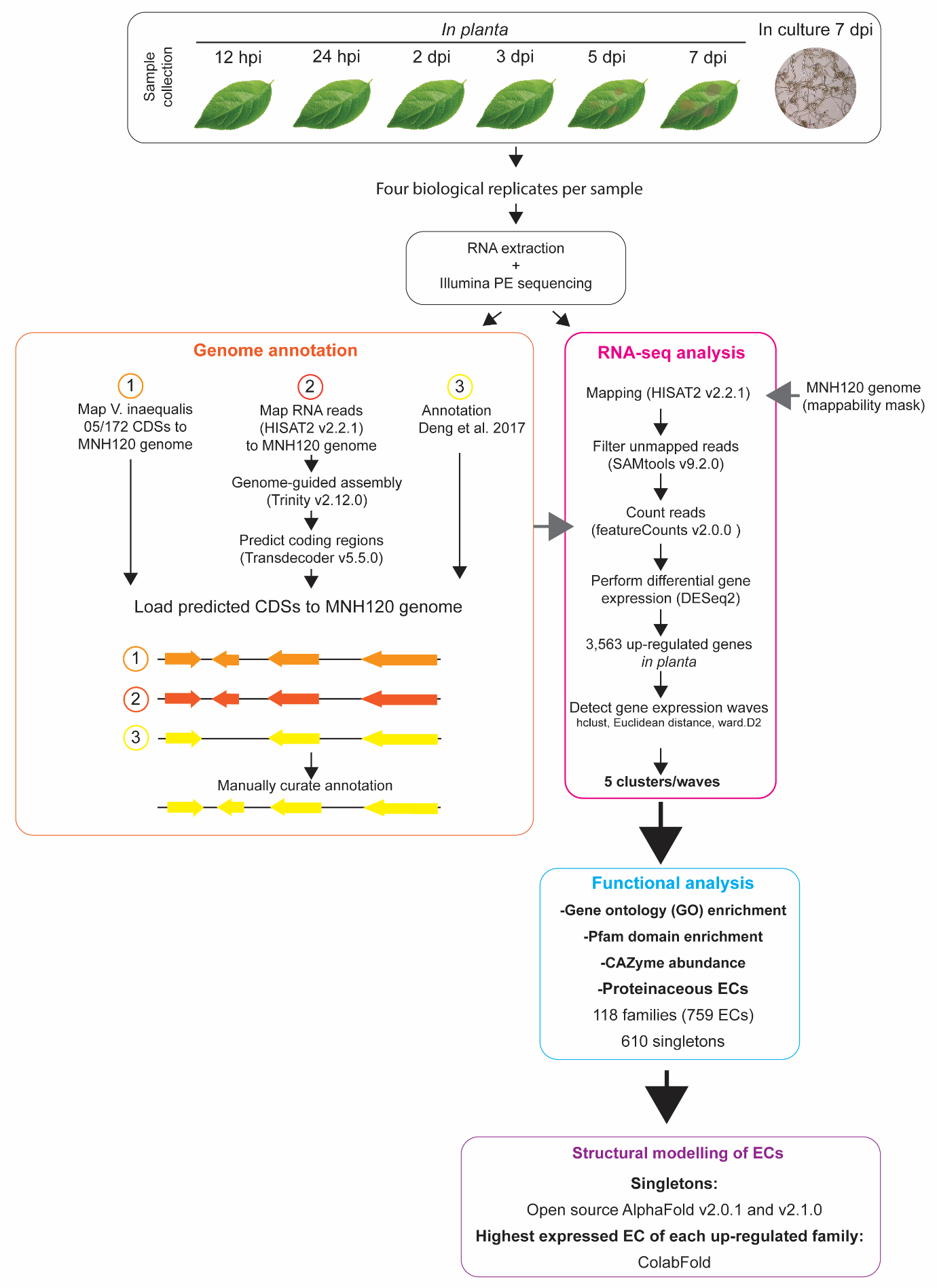


**Fig. S1** Bioinformatic pipeline used for transcriptome analysis and genome annotation. Total RNA was extracted from apple leaves infected with *Venturia inaequalis* at 12 and 24 hours post-inoculation (hpi), as well as 2, 3, 5 and 7 days post-inoculation (dpi). As a reference for growth in culture, total RNA was also extracted from *V. inaequalis* grown on the surface of cellophane membranes overlaying potato dextrose agar at 7 dpi. Four biological replicates were included per sample. PE: paired-end; CDS: coding sequence; CAZyme: carbohydrate-active enzyme; EC: effector candidate.


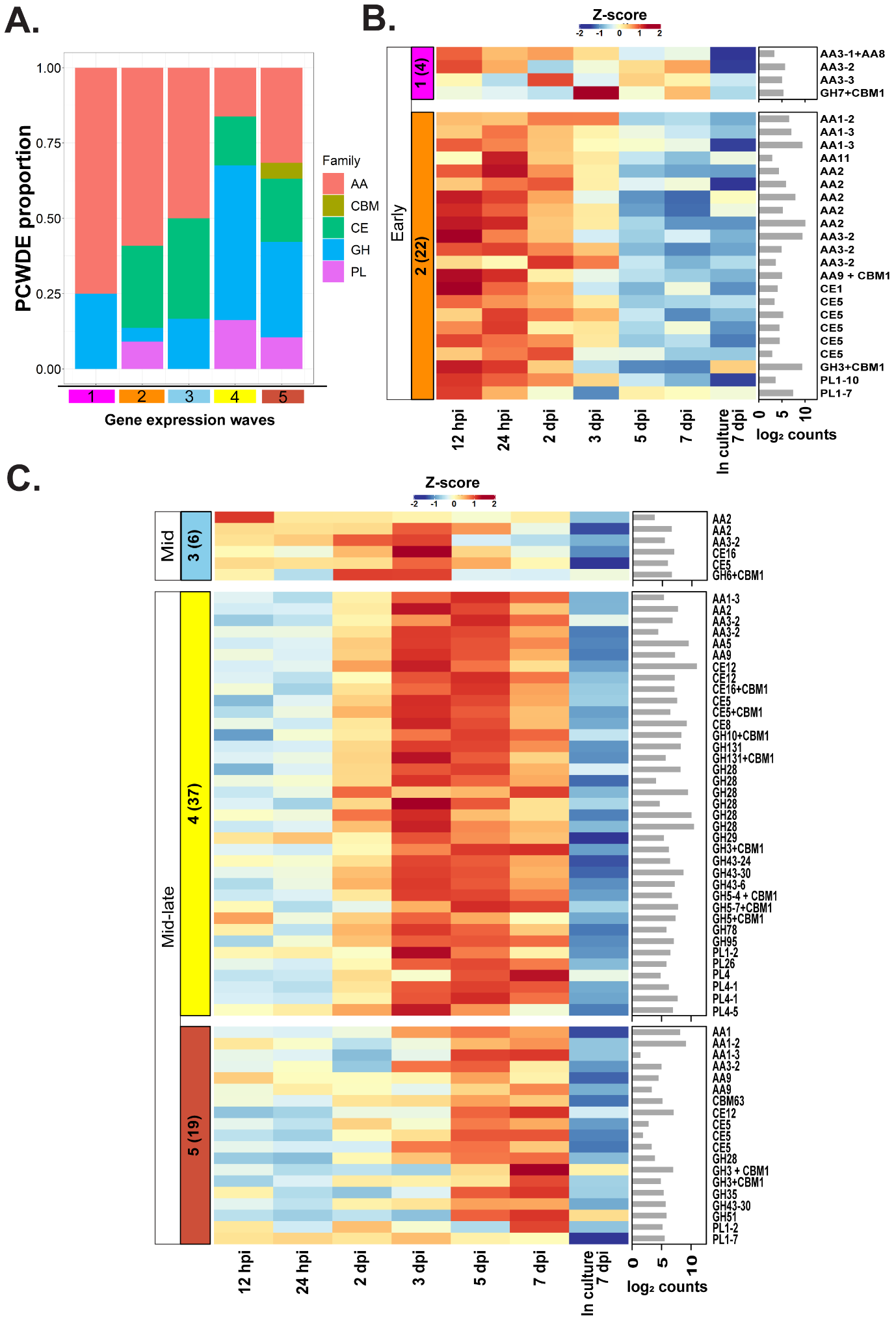


**Fig. S2** Plant cell wall-degrading enzyme (PCWDE)-encoding genes of *Venturia inaequalis* up-regulated during infection of susceptible apple cultivar ‘Royal Gala’, relative to growth of the fungus in culture on the surface of cellophane membranes overlying potato dextrose agar. **A.** Proportion of *in planta* up-regulated PCWDE-encoding genes in each host infection-specific temporal expression wave. **B.** Heatmap of PCWDE-encoding genes up-regulated *in planta* that demonstrate a peak level of expression during waves 1 and 2 of the early infection stage at 12 and 24 hours post-inoculation (hpi). **C.** Heatmap of PCWDE-encoding genes up-regulated *in planta* that demonstrate a peak level of expression during wave 3 of the mid infection stage at 2 and 3 days post-inoculation (dpi) and waves 4 and 5 of the mid-late infection stage at 5 and 7 dpi. Block labels on the left indicate gene expression wave. Numbers in brackets indicate number of genes per wave. Gene expression data are scaled rlog-normalized counts across all samples (Z-score), averaged from four biological replicates. Labels on the right indicate carbohydrate-active enzyme (CAZyme) classification. Bar plots depict the maximum log_2_ DESeq2-normalized count value across all *in planta* time points. AA: auxiliary activity; GH: glycoside hydrolase; CE: carbohydrate esterase; PL: polysaccharide lyase; CBM: carbohydrate-binding module.


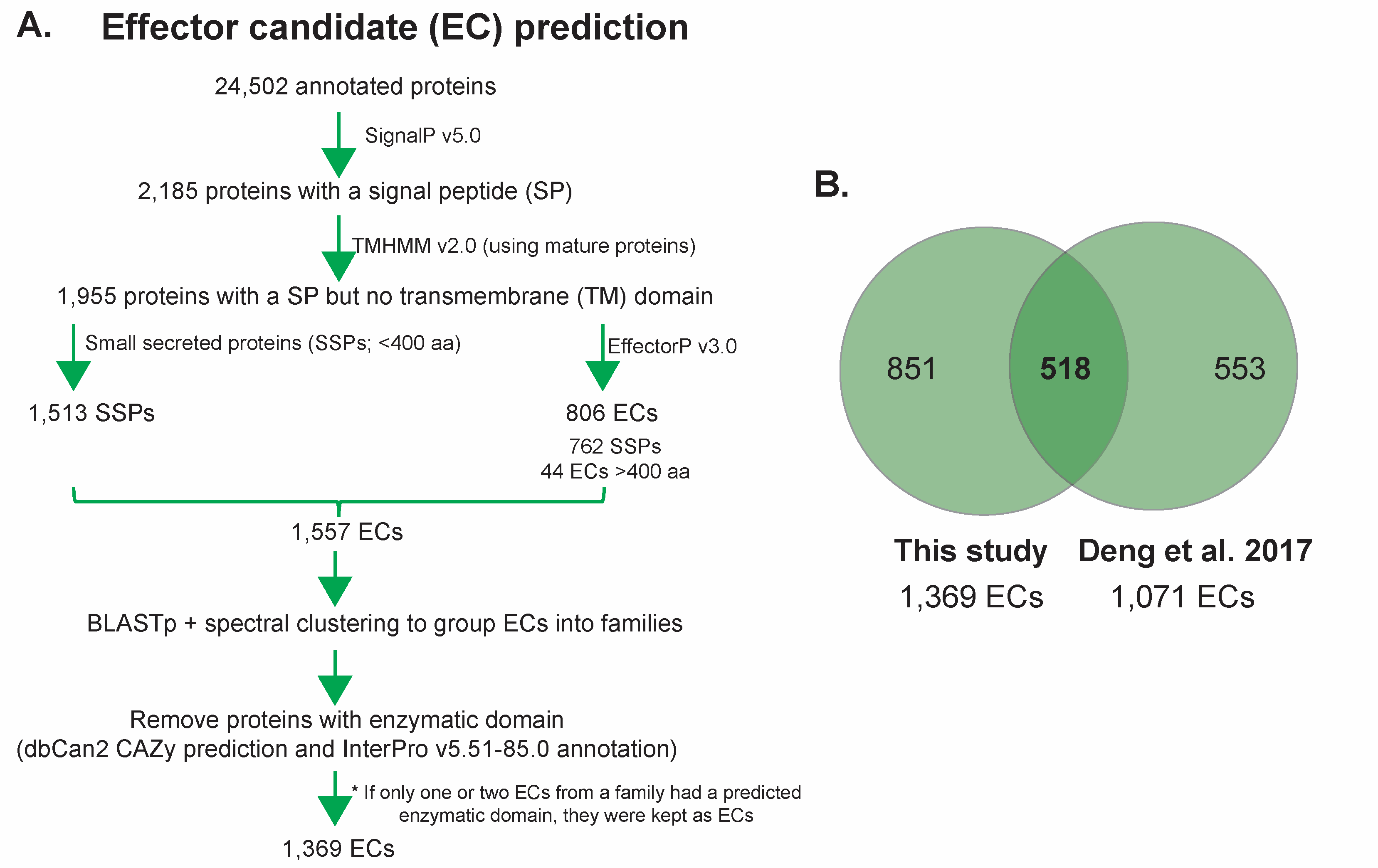


**Fig. S3** Prediction of effectors from *Venturia inaequalis*. **A.** Pipeline for the identification of effector candidates (ECs) from *V. inaequalis****.* B.** Comparison of the number of ECs predicted from *V. inaequalis* isolate MNH120 in this study and a previous study (Deng et al., 2017). The comparison is based on an exact protein sequence match. The previous study by Deng et al. (2017) defined ECs as small proteins of <500 amino acid residues in length with a signal peptide and no similarity to lytic enzymes.


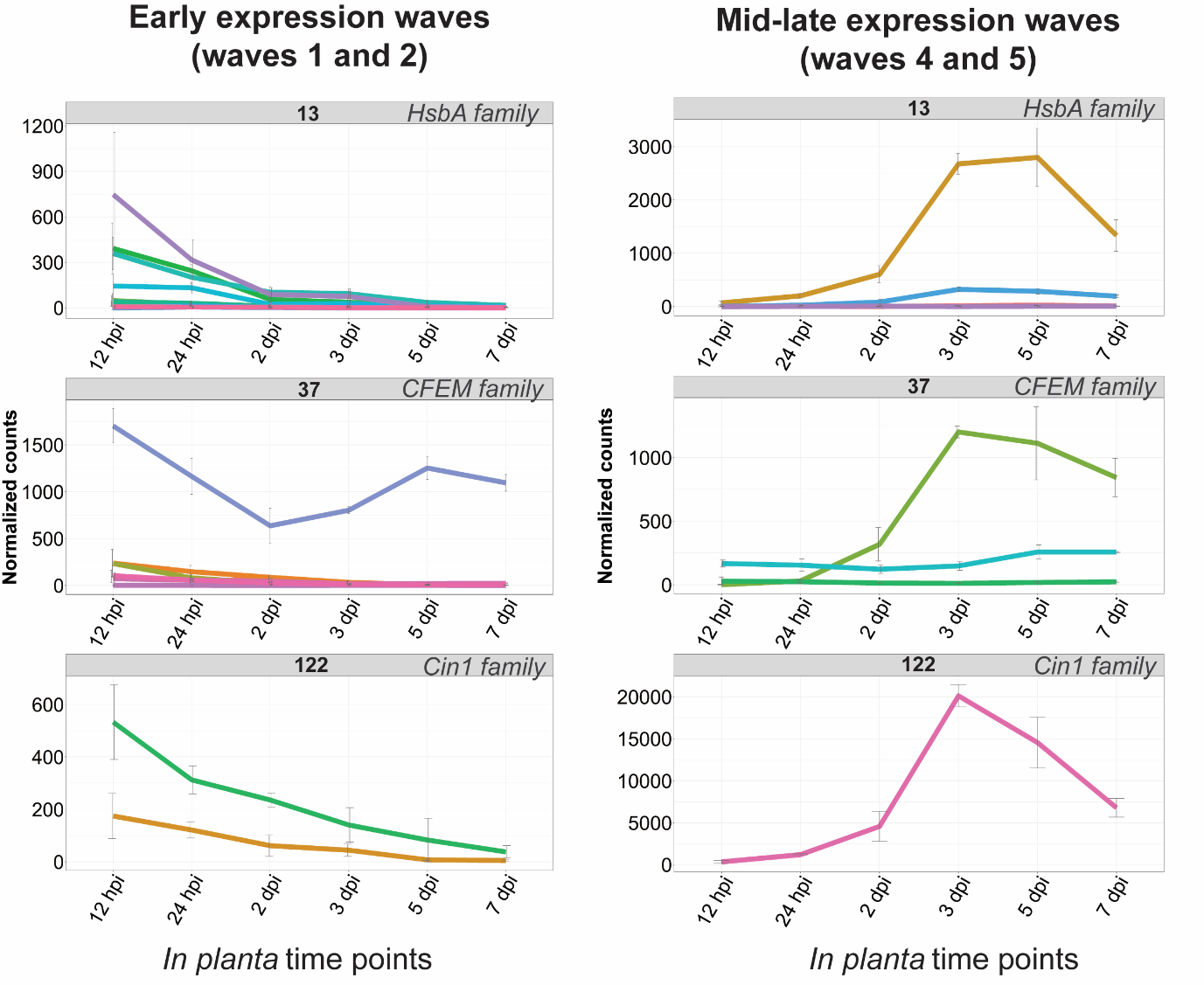
 **Fig. S4** Gene families encoding proteinaceous effector candidates (ECs) of *Venturia inaequalis* that have members demonstrating different expression profiles during early and mid-late colonization of susceptible apple cultivar ‘Royal Gala’. Expression data during host colonization are DESeq2-normalized counts, averaged from four biological replicates, with error bars representing standard deviation (hpi: hours post-inoculation; dpi: days post-inoculation). *HsbA*: Hydrophobic surface-binding protein A; *CFEM*: common fold in several fungal extracellular membrane proteins; *Cin1*: Cellophane-induced 1.


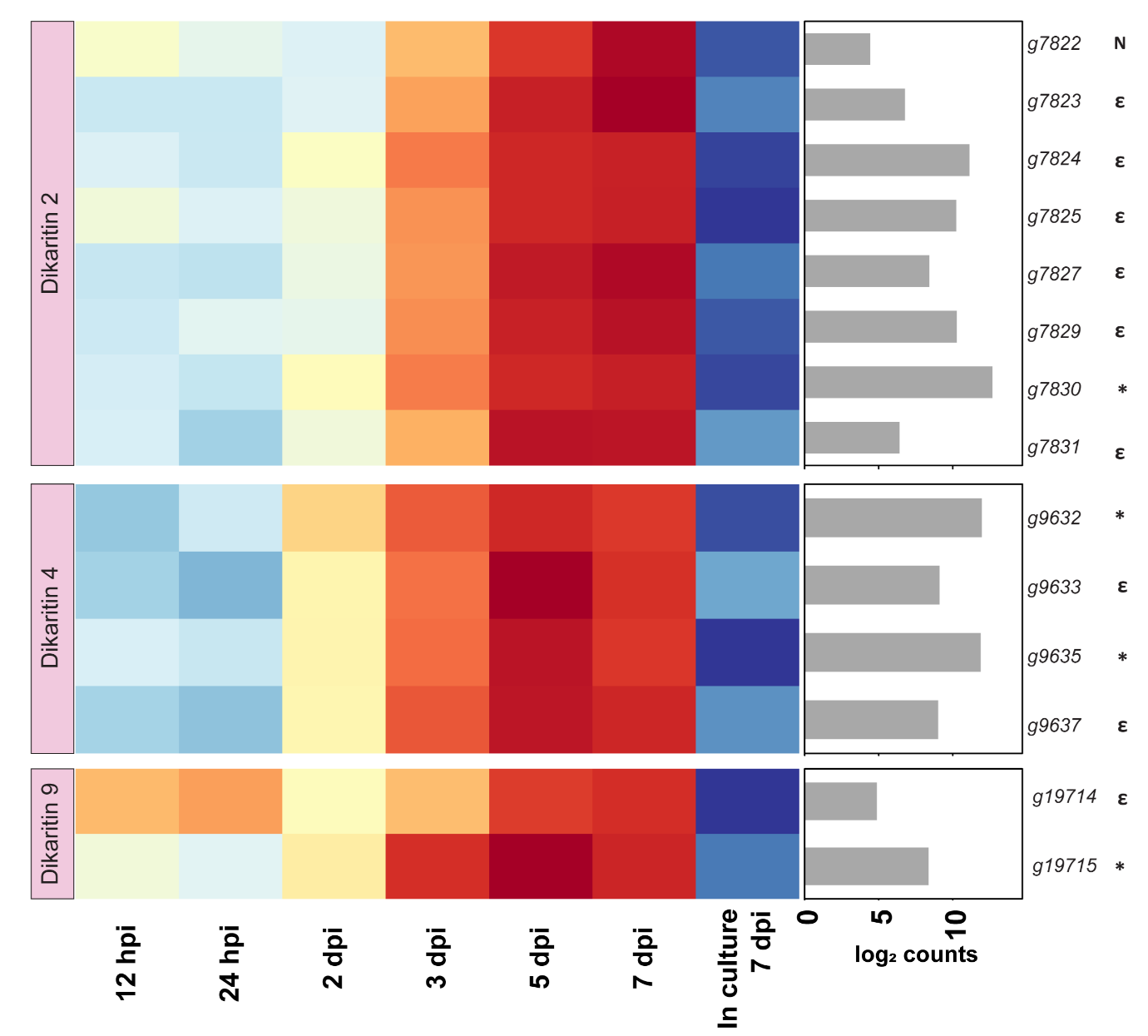
 **Fig. S5** Expression of ribosomally-synthesized and post-translationally modified peptide (*RiPP*) *dikaritin* gene clusters from *Venturia inaequalis* that are up-regulated during colonization of susceptible apple cultivar ‘Royal Gala’, relative to growth of the fungus in culture on the surface of cellophane membranes overlying potato dextrose agar. Heatmap gene expression data are scaled rlog-normalized counts across all samples (Z-score), averaged from four biological replicates. hpi: hours post-inoculation; dpi: days post-inoculation. Bar plot annotation depicts the maximum log_2_ DESeq2-normalized count value across all *in planta* time points. Genes marked as ε putatively encode a protein with a DUF3382 domain or were annotated as a major-facilitator superfamily protein. Genes marked with * putatively encode a dikaritin precursor peptide. The gene marked with N encodes a protein with no characterized functional domain.


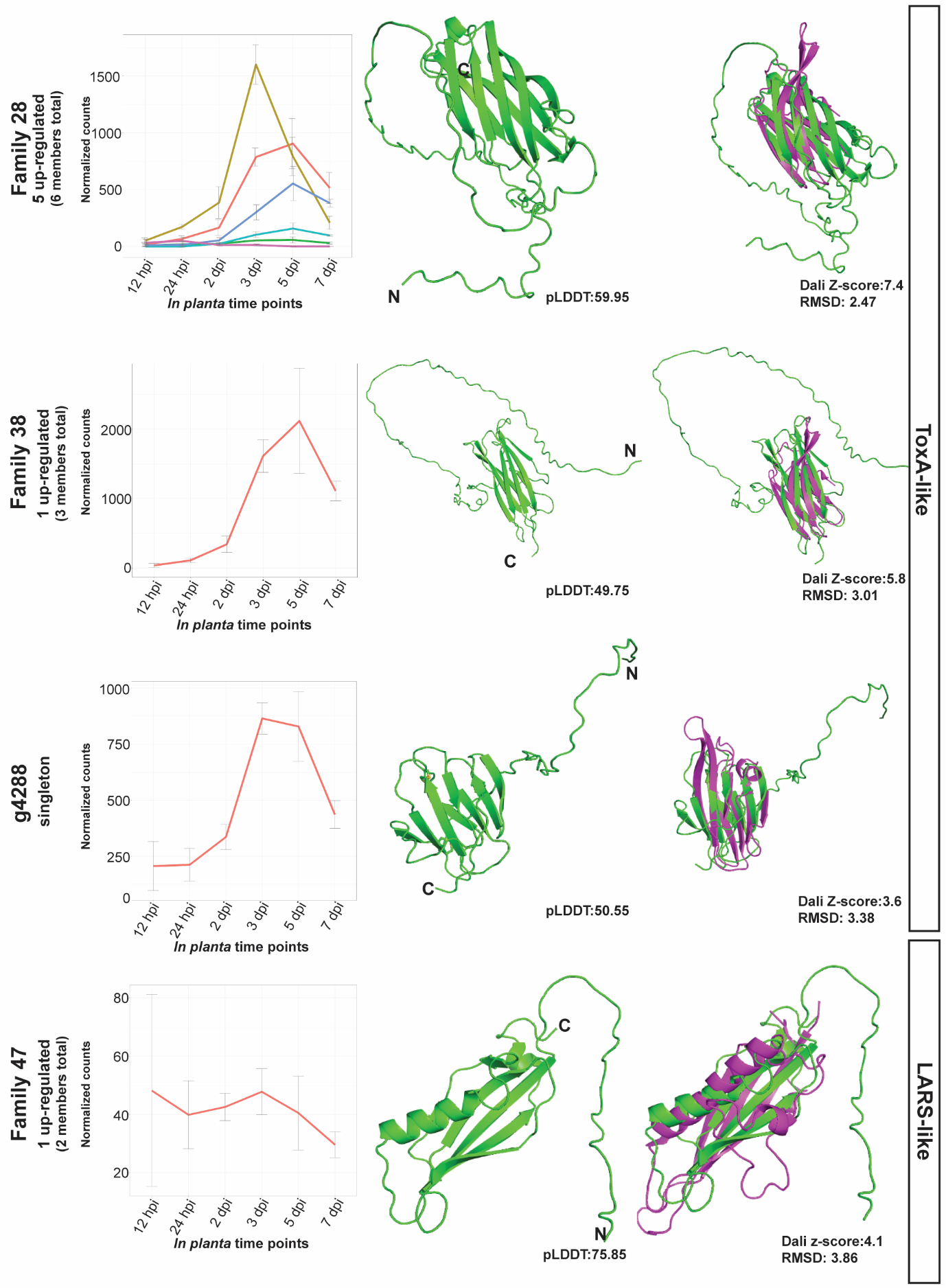


**Fig. S6** Effector candidates (ECs) from *Venturia inaequalis* with structural similarity to known avirulence (Avr) effector proteins from other plant-pathogenic fungi not shown in Fig 4. Representative *V. inaequalis* protein structures (green; family 28 (g13172), family 38 (g9034), singleton g4288) aligned to ToxA from *Pyrenophora tritici-repentis* (1ZLE) (purple), LARS-like protein structure (green; family 47 (g24490)) aligned to AvrLm4-7 from *Leptosphaeria maculans* (7FPR). Protein tertiary structures predicted by AlphaFold2 are the most highly expressed member from each EC family. Disulfide bonds coloured in yellow. N: amino (N) terminus; C: carboxyl (C) terminus. pLDDT: predicted Local Distance Difference Test score (0‒100); a pLDDT score of 70–100 is indicative of medium to high confidence. A Dali Z-score above 2 indicates ‘significant similarities’ between structures. RMSD: root-mean-square deviation. Gene expression data are from up-regulated *ECs* during host colonization and are based on DESeq2-normalized counts, averaged from four biological replicates, with error bars representing standard deviation (hpi: hours post-inoculation; dpi: days post-inoculation).


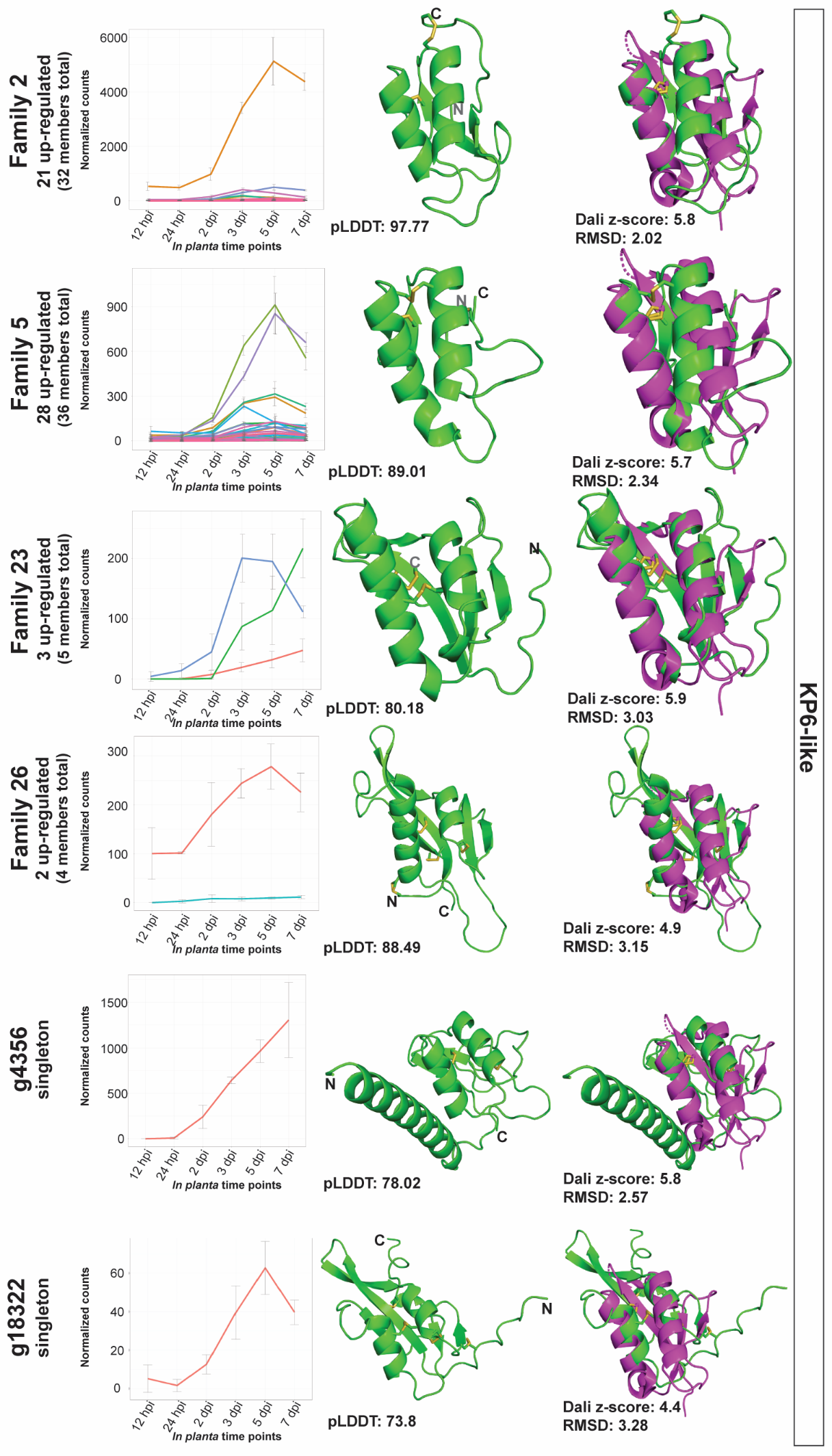


**Fig. S7** Effector candidates (ECs) from *Venturia inaequalis* with structural similarity to killer protein 6 (KP6) from *Ustilago maydis* P6 virus not shown in Fig 4. Representative *V. inaequalis* protein structures (green; family 2 (g11711), family 5 (g18375), family 23 (g4577), family 26 (g12079), singleton g4356, singleton g18322) aligned to EC Zt-KP6-1 from *Zymoseptoria tritici* (6QPK). Protein structures predicted by AlphaFold2 are the most highly expressed member from each EC family. Disulfide bonds coloured in yellow. N: amino (N) terminus; C: carboxyl (C) terminus. pLDDT: predicted Local Distance Difference Test score (0‒100); a pLDDT score of 70–100 is indicative of medium to high confidence. A Dali Z-score above 2 indicates ‘significant similarities’ between structures. RMSD: root-mean-square deviation. Gene expression data are from up-regulated *ECs* during host colonization and are based on DESeq2-normalized counts, averaged from four biological replicates, with error bars representing standard deviation (hpi: hours post-inoculation; dpi: days post-inoculation).


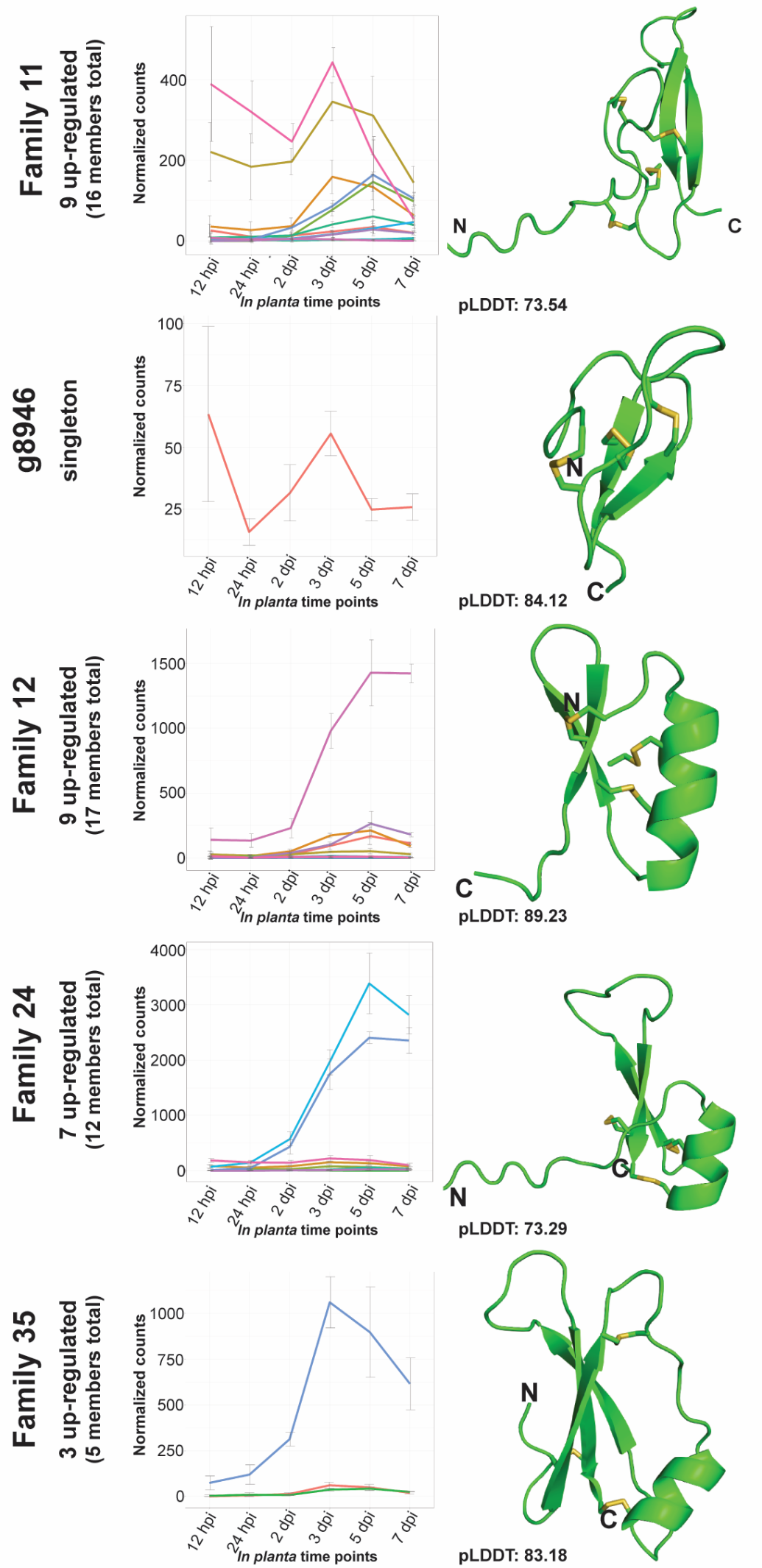


**Fig. S8** Effector candidates (ECs) from *Venturia inaequalis* with a predicted knottin-like fold. Representative *V. inaequalis* structures (green; family 11 (g8686), family 12 (g10808), family 24 (g22936), family 35 (g22623), singleton g8946). Protein structures predicted by AlphaFold2 are the most highly expressed member of each EC family. Disulfide bonds coloured in yellow. N: amino (N) terminus; C: carboxyl (C) terminus. pLDDT: predicted Local Distance Difference Test score (0‒100); a pLDDT score of 70–100 is indicative of medium to high confidence. A Dali Z-score above 2 indicates ‘significant similarities’ between structures. Gene expression data are from up-regulated *ECs* during host colonization are based on DESeq2-normalized counts, averaged from four biological replicates, with error bars representing standard deviation (hpi: hours post-inoculation; dpi: days post-inoculation).


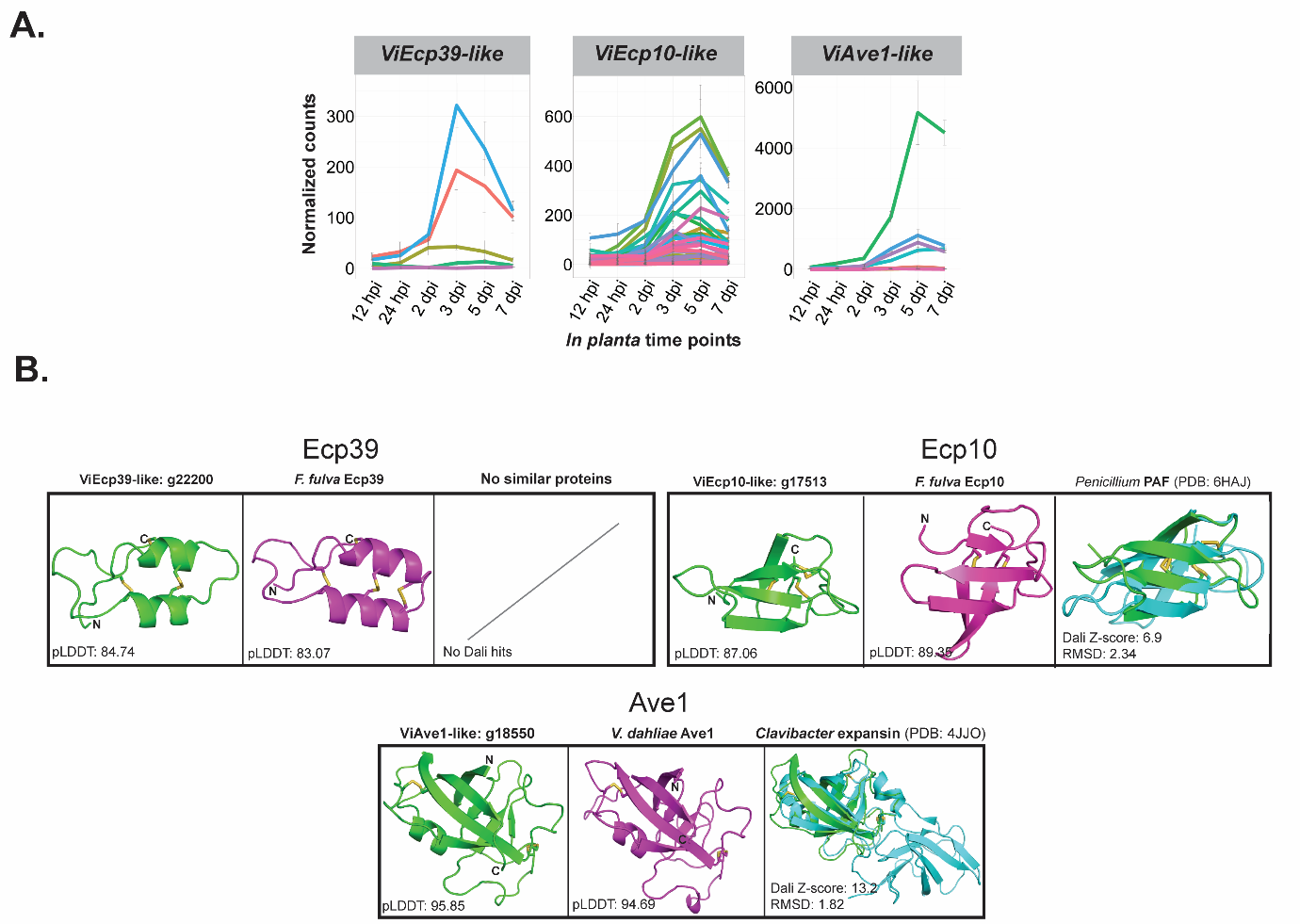


**Fig. S9** Effector candidate (EC) families from *Venturia inaequalis* (*Vi*) with sequence similarity to effectors and characterized or candidate avirulence (Avr) effector proteins from other plant-pathogenic fungi. **A.** Expression data of *EC* genes during host colonization are DESeq2-normalized counts, averaged from four biological replicates, with error bars representing standard deviation (hpi: hours post-inoculation; dpi: days post-inoculation). **B.** Protein tertiary structures of candidate virulence and avirulence (Avr) effector proteins predicted by AlphaFold2. Disulfide bonds coloured in yellow. *F. fulva* is *Fulvia fulva* and *V. dahliae* is *Verticillium dahliae*. Green tertiary structures represent the *V. inaequalis* protein; purple structures represent the EC/Avr from the other fungal pathogen; cyan, represents closest analogous structure in the RCSB PDB database. Sequence similarity was identified by reciprocal protein searches based on BLASTp (E-value <0.05). pLDDT: predicted Local Distance Difference Test score (0‒100). A pLDDT score of 70–100 is indicative of medium to high confidence. A Dali Z-score above 2 indicates ‘significant similarities’ between proteins. RMSD: root-mean-square deviation.


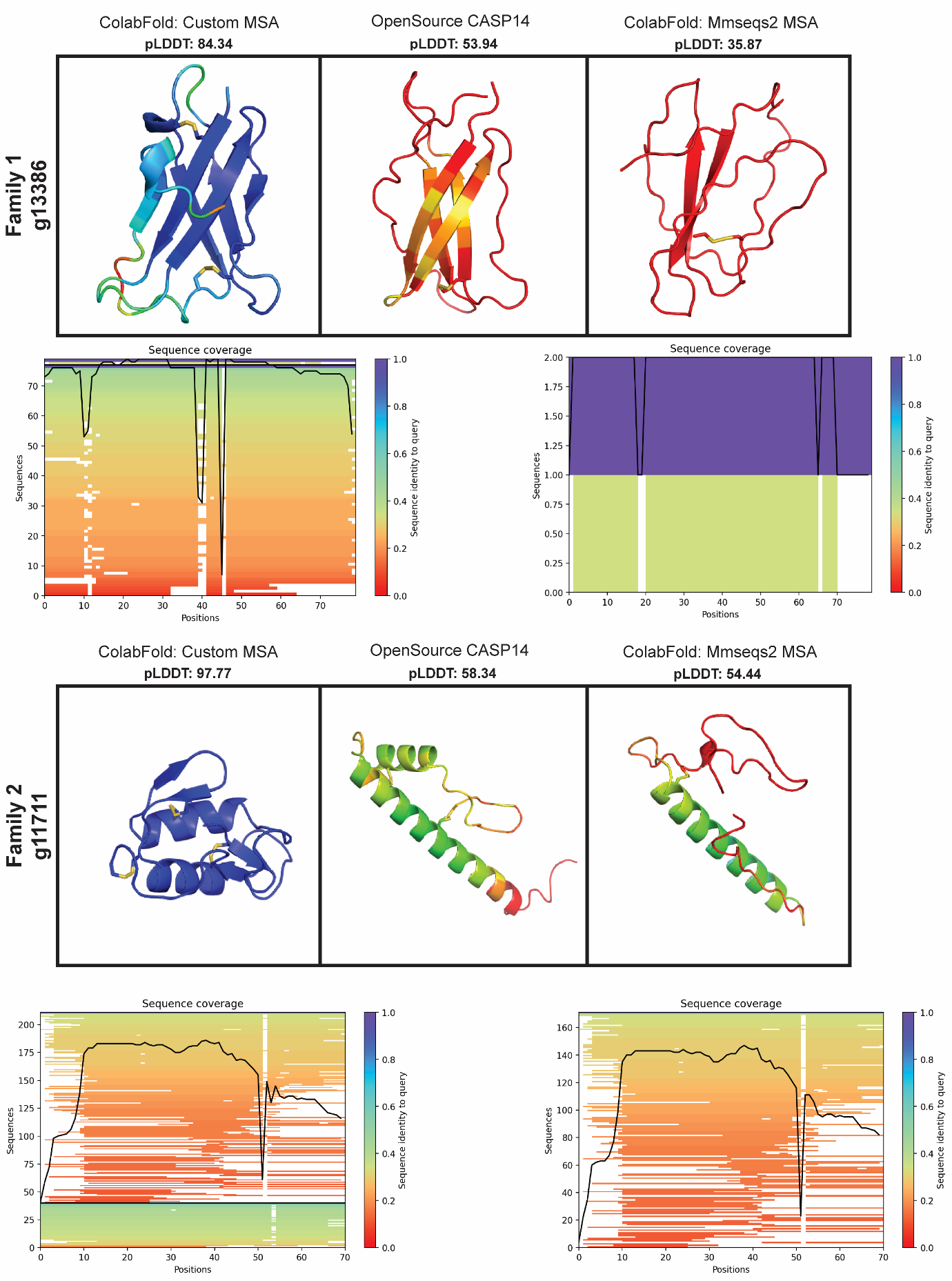


**Fig. S10** Comparison of protein tertiary structures predicted for two effector candidates (ECs) of *Venturia inaequalis* using different AlphaFold2 methods. EC protein tertiary structures are coloured by amino acid pLDDT (predicted Local Distance Difference Test) score, with a high pLDDT score coloured in blue and a low pLDDT score coloured in red. ColabFold multiple sequence alignments (MSAs) were generated with MMseqs2 (Steinegger & Söding, 2017) and MSAs generated by AlphaFold2 open source CASP14 were assembled using HHBlits and HMMER (Johnson et al., 2010; Remmert et al., 2011). Sequence coverage graphs of the MSAs used for the structural predictions were automatically generated by ColabFold (Mirdita et al., 2022).
