# Supplementary material for: The Venturia inaequalis effector repertoire is dominated by expanded families with predicted structural similarity, but unrelated sequence, to avirulence proteins from other plant-pathogenic fungi
Source: BMC Biol. 2022 Nov 3;20:246. doi: 10.1186/s12915-022-01442-9 (PMC9632046; doi:10.1186/s12915-022-01442-9)
Supplement: Supplementary file 8 — Additional file 8: S1 Text. Supplementary methods for the identification of dikaritin RiPP gene clusters in Venturia inaequalis. [file 12915_2022_1442_MOESM8_ESM.docx]

**S1 Text.** Supplementary methods for the identification of *dikaritin* *RiPP* gene clusters in *Venturia inaequalis*.

As a starting point for the identification of *dikaritin* *RiPP* gene clusters in *V. inaequalis*, genes encoding DUF3328 proteins were identified through a tBLASTn analysis of the MNH120 genome in Geneious v9.1.8, using the g7827 DUF3328 protein of *V. inaequalis* (Dikaritin cluster 2) as a query. This was followed by exhaustive reciprocal tBLASTn analyses of the identified sequences against the *V. inaequalis* genome until no new sequences were identified. The resulting protein sequences were then analysed using InterproScan v5.51-85.0 to identify those that carried a putative DUF3328 domain [1]. Notably, some proteins were not predicted to contain a DUF3328 domain or were instead predicted to belong to the Major-facilitator superfamily. These proteins were manually investigated and proteins with a characteristic DUF3328 domain structure, consisting of a short amino (N)-terminal tail (34–58 aa), a transmembrane (TM) domain (19–24 aa) and a domain containing the tandem HxxCH DUF3328 active site motif, were manually annotated as putative DUF3328 proteins. Up to 10 genes up- and down-stream of the putative DUF3328-encoding gene in the MNH120 genome were then assessed for signatures of a classical dikaritin RiPP precursor-encoding gene (i.e. a gene that encodes a precursor peptide with an N-terminal signal peptide, followed by one or more perfect or imperfect tandem sequence repeats of at least 10 amino acids in length that are separated by putative kexin protease cleavage sites), and analysed for functional domains using InterproScan v5.51-85.0. Finally, to define the borders of *dikaritin* *RiPP* gene clusters, transcriptome data from this study were used to investigate the expression of genes encoding the DUF3328 domain-containing protein and putative precursor protein, as well as the surrounding genes. Only those genes with a similar *in planta* expression profile to the genes encoding the DUF3328 domain-containing protein and putative precursor peptide were considered to form part of the *dikaritin* *RiPP* gene cluster in question.

**Reference**

1. Jones P, Binns D, Chang H-Y, Fraser M, Li W, McAnulla C, McWilliam H, Maslen J, Mitchell A, Nuka G *et al*: InterProScan 5: genome-scale protein function classification. *Bioinformatics* 2014, 30(9):1236-40.
